# The Detection and Surveillance of Asian Citrus Psyllid (*Diaphorina citri*)—Associated Viruses in Florida Citrus Groves

**DOI:** 10.3389/fpls.2019.01687

**Published:** 2020-01-17

**Authors:** Kellee Britt, Samantha Gebben, Amit Levy, Maher Al Rwahnih, Ozgur Batuman

**Affiliations:** ^1^Department of Plant Pathology, Southwest Florida Research and Education Center, University of Florida, Immokalee, FL, United States; ^2^Department of Plant Pathology, Citrus Research and Education Center, University of Florida, Lake Alfred, FL, United States; ^3^Department of Plant Pathology, University of California-Davis, Davis, CA, United States

**Keywords:** Asian citrus psyllid, insect viruses, Candidatus Liberibacter asiaticus, Huanglongbing, biological control

## Abstract

The plant pathogenic bacterium *Candidatus* Liberibacter asiaticus (*C*Las), the causal agent of the citrus disease Huanglongbing (HLB), and its insect vector, the Asian citrus psyllid (ACP; *Diaphorina citri*), have been devastating the Florida citrus industry. To restore the competitive production presence of Florida in the worldwide citrus market, effective and sustainable control of HLB and the ACP needs to be identified. As alternatives for resistance-inducing insecticides, viruses are currently being considered for biological control of the ACP. To identify possible biological control candidates, we conducted one of the most comprehensive surveys of natural ACP populations in major citrus production regions spanning 21 counties in Florida. By optimizing PCRs and RT-PCRs, we were able to successfully detect and monitor the prevalence of five previously identified ACP-associated RNA and DNA viruses throughout Florida citrus groves, which include: *Diaphorina citri-associated C virus* (DcACV), *Diaphorina citri flavi-like virus* (DcFLV), *Diaphorina citri densovirus* (DcDNV)*, Diaphorina citri reovirus* (DcRV), and *Diaphorina citri picorna*-*like virus* (DcPLV). Adult and nymph ACP populations from 21 of Florida's major citrus-producing counties were collected each month during approximately 18 consecutive months. RNA extracts used for these viral screens were also regionally combined and subjected to High Throughput Sequencing (HTS) to reveal a more comprehensive picture of known and unknown viruses in Florida ACP populations. We discovered that DcACV was the most prevalent ACP-associated virus throughout nymph and adult ACP populations in Florida, detected in more than 60% of all samples tested, followed by DcPLV and DcFLV. HTS allowed us to identify a novel ACP-associated reo-like virus and a picorna-like virus. The putative reo-like virus, tentatively named *Diaphorina citri* cimodo-like virus, was later surveyed and detected back in seasonal adult and nymph ACP samples collected in Florida during this study. HTS generated data also revealed that the most abundant virus in Florida ACP populations was *Citrus tristeza virus* (CTV), which is not an ACP-associated virus, suggesting persistent presence of CTV infection in citrus throughout Florida groves. Collectively, information obtained from our study may be able to help guide the direction of biotechnological pest control efforts involving a number of viruses that were detected for the first time in Florida ACP populations, including two newly identified ACP-associated viruses.

## Introduction

The bacterial pathogen, *Candidatus* Liberibacter asiaticus (*C*Las), the causal agent of the citrus disease Huanglongbing (HLB), continues to endanger the worldwide citrus industry, with the most severe losses occurring in Florida (Jagoueix et al., 1994; Garnier and Bové, 1996; do Carmo Teixeira et al., 2005). Currently, this disease threatens the future of Florida's $9 billion citrus industry and simultaneously threatens production sites in Texas and California (Gottwald, 2010; Hall et al., 2013). The fastidious, phloem limited *C*Las bacterium cannot be cultured, preventing the completion of Koch's postulates and *in vitro* biological studies (Jagoueix et al., 1996; do Carmo Teixeira et al., 2005). Although short-term disease management using bactericides, plant immune activators, nutritional supplements, and heat treatments have been able to slightly reduce the acceleration of the citrus plant decline, the HLB pathogen shows vigorous strength against them and eventually overcomes these temporary therapies (Li et al., 2016; Blaustein et al., 2018; Munir et al., 2018).

The insect vector of this bacterium, the Asian citrus psyllid (ACP; *Diaphorina citri* Kuwayama), has been proven to be the primary means of dispersal of this disease throughout citrus groves in Florida (Hall et al., 2013). It has also been shown that each feeding event contributes substantially to the development of disease severity and on the ultimate survival of the infected trees (Stansly et al., 2014; Lee et al., 2015). ACP control is therefore essential in Florida citrus groves and the growers' investment in vector control could still have considerable effect on the production of their trees, even with *C*Las infection (Stansly et al., 2014). Current HLB disease surveys of Florida citrus groves show approximately 95% of trees are symptomatic, with no signs of decreasing incidence. Management options for HLB are limited and rely heavily on insecticides for controlling ACP populations, even when integrated with various cultural control methods (Grafton-Cardwell et al., 2013; Blaustein et al., 2018). Unfortunately, these chemical strategies are mostly ineffective due to challenges associated with repetitive and expensive applications and the inability to consistently decrease ACP populations. Growers are also concerned with the development of chemical resistance among ACP populations and the threat long-term chemical applications pose to the environment and beneficial organisms (Tiwari et al., 2011; Hall et al., 2013; Kanga et al., 2016; Chen et al., 2017; Chen and Stelinski, 2017; Pardo et al., 2018). To prevent new citrus plantings from HLB infection and to avoid the development of pesticide-resistant ACP populations, many researchers are targeting control of the vector through biological means (Chen et al., 2018; Tian et al., 2018).

As the most abundant entity on the planet and in the ACP, viruses offer limitless molecular and biotechnological opportunities to explore and can provide further knowledge of the *C*Las-vector interaction. Virus-induced gene silencing (VIGS), which has been documented as an effective way to deliver RNA interference (RNAi) into both plants and insects, is a molecular strategy for the biological control of the ACP and eventual disruption of the HLB disease cycle (Baulcombe, 1999; Hall et al., 2013; Nandety et al., 2015; Joga et al., 2016; Galdeano et al., 2017). Another phloem-limited citrus pathogen, *Citrus tristeza virus* (CTV), is currently being used as a viral vector to deliver this RNAi technology into the citrus phloem and then into the ACPs that feed on citrus (Hajeri et al., 2014; Killiny et al., 2019). However, these routes can be cumbersome, require an established infection in the citrus plant before delivering RNAi to the insect, and may target only a few insect organs other than the gut. Employing an endogenous ACP-associated virus that may be naturally replicating and expressing its proteins in the insect can have the potential to deliver RNAi with higher efficiency and lower chances of deleterious, off-target effects.

As a viable alternative to chemical controls, development of knowledge-based and efficient biological control strategies to disrupt *C*Las transmission by current technologies (i.e., RNAi) in the ACP vector itself represent our strategy to control the disease without relying solely on chemical applications. The necessity of a precise and specific biological control for Florida ACP populations initiated this study, with hopes to provide additional molecular tools. In order to decrease or eliminate major dependence on broad-spectrum chemical controls, this study aimed to investigate the Florida ACP virome for identifying an ACP-associated virus with the potential to be manipulated into a biocontrol agent against the HLB insect vector. The endemic establishment of both HLB and the ACP in Florida has greatly increased the need for alternative control options.

Prior to this study, Nouri et al. (2016a) used metagenomic analysis in a limited number of worldwide ACP samples and identified five new viruses, with the assemblage of nearly complete viral genome sequences (Nouri et al., 2016a; Nouri et al., 2016b). These included several putative novel viruses associated with *D. citri*, such as a picorna-like virus, a reovirus, a densovirus, a bunyavirus, and an unclassified (+) ssRNA virus (Nouri et al., 2016a; Nouri et al., 2018).

The Nouri et al. (2016a) study and our initial observations of various viral particles in the ACP nymphs and adults *via* transmission electron microscopy (TEM) (unpublished data) lead us to our current strategy for identifying and detecting ACP-associated viruses, especially in Florida ACP populations. Additionally, *C*Las survival, reproduction, and dissemination has been observed to occur principally in the gut of the ACP, which has influenced supplementary targeted approaches for potentially inhibiting bacterial persistence in this organ (Ammar et al., 2011; Ghanim et al., 2016; Ghanim et al., 2017). It was observed that apoptosis, an ACP defense response to microbial invasion, was activated to target and destroy (or limit) the *C*Las infection and spread. Ghanim et al. (2017) documented the occurrence of several upstream events leading to these apoptotic responses, which commenced in the endoplasmic reticulum (ER) of the ACP gut. In addition, the same study provided direct evidence of *C*Las replication in the midgut cells of ACP, which occurs inside Liberibacter-containing vacuoles and associates with the gut ER (Ghanim et al., 2017). Thus, an unknown virus in the gut, if manipulated, could serve as a highly efficient and selective biological control agent for ACP when consumed by the ACP and initiated our search for this virus as a viable ACP biocontrol option. It is still unknown whether these viruses are beneficial or destructive to ACPs; however, their presence in ACP populations around the world suggests the likelihood of high persistence and may therefore be a conceivable vector for RNAi delivery. Through this study, which we believe is the most comprehensive survey in natural ACP populations (i.e., not laboratory-reared) in Florida, we intend to further characterize the ACP virome as it relates to Florida citrus groves, where the HLB epidemic has infiltrated and caused unprecedented economic damage. This may bring us closer to identifying the unknown ACP-associated virus localized in the gut. We believe the future of biological control using manipulated ACP-associated viral vectors for RNAi delivery will involve targeting and blocking an essential gene in the insect to control HLB.

The main objectives of this study include surveying and monitoring previously characterized ACP-associated viruses in nymph and adult ACP populations in Florida citrus groves with PCR-based methods and simultaneously subjecting subsamples of these populations to High Throughput Sequencing (HTS) technologies. The first objective will provide foundational knowledge of the known viruses that might already be present in Florida ACP populations, with regards to their spatial and temporal characteristics throughout citrus-producing counties. The second objective will provide a more thorough analysis of the Florida ACP virome, including the detection of novel and other known viruses. By analyzing ACPs from around the world, Nouri et al. (2016a) had identified and characterized new ACP viruses from a small number of psyllid colonies; however, we've concentrated our samples in this study toward larger and naturally occurring ACP populations collected solely from Florida citrus groves and focused our efforts on discovering or identifying other robust and novel Florida ACP viruses. Results of this study may uncover a better candidate for an alternative viral vector used for delivering RNAi technology to combat the ACP in Florida.

## Materials and Methods

### ACP Collection and Processing

Adult and nymph ACPs were collected directly from citrus trees in commercial groves in 21 of Florida's major citrus-producing counties around south-central Florida every month over a period of approximately eighteen months (August 2017 through December 2018). One grove, ranging from 500 to 2,000 acres (~200 to 800 hectare), was sampled per county and samples were taken randomly from multiple trees in one block of each grove usually around the perimeter of the block, where the observation of ACP incidence has been the greatest (Sétamou and Bartels, 2015). Each citrus grove sample site, which represented each county, remained consistent throughout the entire study, except for a few groves that closed or changed operation during the months of our sampling. These sites were immediately replaced with another grove site within the same county.

Young citrus shoots with adult or nymph ACPs were carefully detached from trees and immediately submerged into 50 ml Falcon tubes containing 100% ethanol, killing the ACPs, and the Falcon tubes were kept closed. Since HLB and ACPs are currently widespread in Florida, traveling throughout Florida with (dead) ACPs was not a concern. The number of ACPs collected varied by grove; however, we aimed to collect a minimum of 25 adults and 25 nymph ACPs from each grove every month. There were times in which lower numbers were collected due to inhospitable conditions, such as cooler or rainy weather, or increased pesticide applications. ACPs collected from these 21 groves were brought to the laboratory at the Southwest Florida Research and Education Center (SWFREC) in Immokalee FL, where adult and nymph ACPs were separated out from plant parts, counted with the aid of a stereo microscope (Leica S8 APO, Leica Microsystems, USA), and stored separately in smaller vials containing 100% ethanol at -20°C until needed. Total ACP counts per grove site (representing one county) were then calculated and combined into six regions for data analysis (**Table 1**).

**Table 1:** Total number of ACPs collected in each Florida region over a period of 18 months.

Regions	Number of groves/counties	Total number of samples	Adults	Nymphs	Total
Region A	4	124	1,933	14,053	15,986
Region B	3	72	836	4,535	5,371
Region C	5	150	1,987	15,187	17,174
Region D	4	115	884	8,104	8,988
Region E	4	99	1,578	13,801	15,379
Region F	1	61	876	3,651	4,527

### Total RNA Extraction and cDNA Synthesis

For each monthly-collected sample representing a county, a subset of five adult ACPs and five 5^th^-instar nymph ACPs were randomly selected and separately used for RNA extraction. This was intended to get a snapshot of the viral content (virome) of the psyllid population in each county and each month. Preliminary tests (data not shown) and previous research together determined that five insects of this size (2–5 mm) was sufficient to yield enough total RNA for RT-PCR screenings (Runckel et al., 2011). To verify total RNA extraction and cDNA quality of ACP samples, primers were designed to target the *Diaphorina citri* (*Dc*) wingless gene as an internal control (**Table 2**). Five insects (nymphs or adults) were placed into sterile 1.5 ml Eppendorf tubes with three 2.3 mm chrome steel beads (BioSpec Products Inc, Bartlesville, OK, USA) and sealed with Breathe-Easy tube membranes (Genesee Scientific, CA, USA). All samples were then placed into a Labconco FreeZone 6 Freeze-Dry System (Kansas City, MO, USA) overnight and ground in a Mini Beadbeater™ (BioSpec Products Inc, Bartlesville, OK, USA) to a fine powder. Total RNA was extracted using the Quick-RNA MiniPrep kit (Zymo Research, Irvine, CA, USA) according to the manufacturer's instructions, excluding the DNase treatment in order to screen for DNA viruses in the sample. RNA was stored at -20°C until needed.

**Table 2:** List of primers, gene targets, and amplicon lengths generated to detect the presence of each ACP-associated virus in all samples.

Virus	Primers	Gene target	Length of amplicon
**DcACV**	F: 5' GCCGCACGAAACTAGTGATAAACGCA 3' R: 5' GGATCGGTGGTGCACGAGTATGTAAGTA 3'	RNA1¹ segment	473 bp
**DcFLV**	F: 5' AGGCGAGTACTCCCATCGGATACATT 3' R: 5' GAGGGCCGCTAAGTCTGTAGGACATATT 3'	RdRp²	~1.4 kb
**DcDNV**	F: 5' AGTCGGTGAGACTGATATCTTCGAGACC 3' R: 5' GTTTAGTTCGCTTGTCGGTTACACAGG 3'	RdRp³	~1 kb
**DcRV**	F: 5' TTTTCCCAGGTACATCGA 3' R: 5' ACCATTCAGCCAGTCCTA 3'	p8⁴	900 bp
**DcPLV**	F: 5' TAGGTGAACGTGATAATCCTGGTAT 3' R: 5' CAGAACGTCTGTTATGAATCGGAC 3'	Polyprotein⁵	698 bp
**DcCLV 5'-end**	F: 5' AACACCCATGCTTCCAAAAC 3' R: 5' TCGTTTCACTAACGCCAATTT 3'	Predicted RdRp^6^	492 bp
**DcCLV 3'-end**	F: 5' ATTTAGGGCCATGTGCAAAG 3' R: 5' CCAACACACCGAGCATACAC 3'	Predicted RdRp^6^	526 bp
**Internal control for integrity of ACP cDNA**	F: 5' TCCAGAGTGATGGTCAGTAA 3' R: 5' GATCTCCTGTGTTCTGTAGC 3'	ACP (*Dc*) wingless gene^7^	273 bp

^1^Accession number: KX235518.1; ^2^Accession number: KX267823.1; ^3^Accession number: KX165268; ^4^ Nouri et al, 2016a; ^5^Accession number: KT698837.1; ^6^PCR primers designed for novel candidate virus from high throughput sequencing (HTS) results and used for screening of ACP populations in Florida; ^7^Accession number: XM_008486571.2. DcACV, Diaphorina citri associated C virus; DcFLV, Diaphorina citri flavi-like virus; DcDNV, Diaphorina citri densovirus; DcRV, Diaphorina citri reovirus; DcPLV, Diaphorina citri picorna-like virus; DcCLV, Diaphorina citri cimodo-like virus; F, Forward Primer; R, Reverse Primer; RdRp, RNA-dependent RNA polymerase; bp, base pair; kb, kilobase. An internal control was also used to verify the integrity of RNA extraction and cDNA synthesis steps.

Six microliters of extracted total RNA from ACP and 1.5 μl of a random hexamer primer (250 ng/μl) (Thermo Fisher Scientific, Waltham, MA, USA) were mixed and incubated at 65°C for 5 min followed by incubation on ice for 5 min to begin complementary DNA (cDNA) synthesis. One microliter of Superscript II (200 u) reverse transcriptase (Invitrogen, Carlsbad, CA, USA), 1 μl of dNTPs (10 mM), 2 μl of DTT (0.1 mM) (Thermo Fisher Scientific, Waltham, MA, USA), 4 μl of buffer (5X) (Thermo Fisher Scientific, Waltham, MA, USA) and 4.5 μl of RNAse-free H_2_O were added in a total reaction mix of 20 μl and incubated at 42°C for 1 h. The cDNA was then heated to 65°C for 15 min to deactivate Superscript II. The cDNA was either used immediately in RT-PCR or stored at -20°C until needed.

### ACP-Associated Virus Screenings With RT-PCR and PCR

The cDNA and total RNA from all samples were used to screen all monthly-collected county samples for four previously identified ACP-associated RNA viruses and one DNA virus: *Diaphorina citri-associated C virus* (DcACV), *Diaphorina citri flavi-like virus* (DcFLV), *Diaphorina citri picorna-like virus* (DcPLV), *Diaphorina citri reovirus* (DcRV), and *Diaphorina citri densovirus* (DcDNV), respectively (Marutani-Hert et al., 2009; Matsumura et al., 2016; Nigg et al., 2016; Nouri et al., 2016a; Nouri et al., 2016b). These five viruses were pursued further in this study because of their previous detection in ACP populations (Marutani-Hert et al., 2009; Nouri et al., 2016a). An internal control for ACP cDNA, ACP (*Dc*) wingless gene, was also used in order to verify the integrity of the RNA for each sample. The PCR primers used for each virus, along with the target gene and the length of the amplicon, are listed in **Table 2**. All PCRs were performed using DreamTaq Green (2X) Master Mix (Thermo Fisher Scientific, Waltham, MA, USA) in a reaction volume of 25 μl. The final concentration of each primer was 0.5 μM. The PCR cycles for each ACP-associated virus consisted of a 2-min initial denaturation at 94°C, followed by 35 cycles of 94°C for 20 s, 50°C (DcACV and DcRV), 62°C (DcDNV, DcFLV, and DcPLV), and 60°C (ACP [*Dc*] wingless gene) for 20 s and 72°C for 2 min, with final extension at 72°C for 10 min. PCR products were separated on 1% agarose gels and visualized by staining with Apex™ Safe DNA Stain (Genesee Scientific, San Diego, CA, USA). Randomly selected amplicons with expected size generated from genomic DNA (DcDNV) and cDNA (DcACV, DcFLV, DcPLV, and DcRV), and from corresponding positive control plasmids (kindly provided by Bryce Falk, UC Davis) were purified using the DNA Clean and Concentrate kit (Zymo Research, Irvine, CA, USA). The purified DNA products were sent for Sanger sequencing (MCLAB, South San Francisco, CA, USA) and generated sequences were subjected to the National Center for Biotechnology Information Basic Local Alignment Search Tool (NCBI BLAST) program to validate the target amplicons' identity.

### Total RNA Preparation and HTS

**Figure 1:**
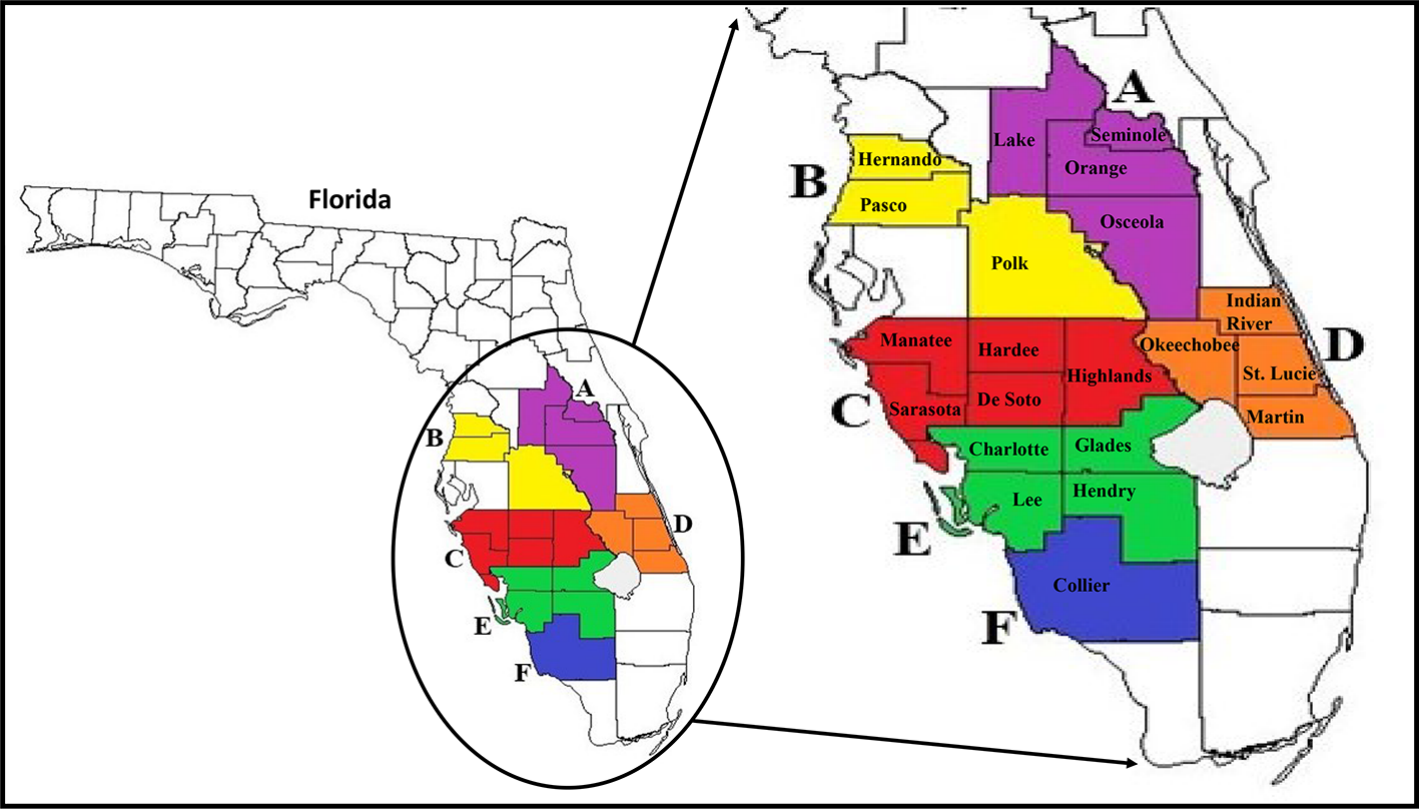
Map of Florida and major citrus production areas (circled) where surveys were conducted in the state. Enlarged map on the right shows the 21 counties (coloured jigsaws with county name) where ACP populations were collected from and the six larger Florida regions (consisting of counties with the same color; **A**–**F**) established for use in all analyses. ACP, Asian citrus psyllid.

The six larger Florida regions (Regions A–F), as depicted in **Figure 1**, were established to group all smaller county samples, which will be used in all future analyses of the study. However, based on our increased access to high numbers of ACPs in Collier County specifically, this county was separately analyzed throughout the study and referred to as Region F. Total RNAs for HTS analysis were extracted from ACP samples using TRIzol (Thermo Fisher Scientific, Waltham, MA, USA) according to the manufacturer's instructions. For HTS, each regional sample consisted of pools of five whole insects (nymphs or adult ACPs) from monthly-collected county samples from early months of the study (August 2017–May 2018) that showed variation in viral incidences in the viral screening methods described above. Total RNAs extracted from each county sample were measured and verified of sufficient quality and purity for HTS using a Synergy HTX plate reader (BioTek Instruments, Winooski, VT, USA) and then combined into the six regional samples.

All six RNA samples were sent to Foundation Plant Services at UC Davis, CA, USA. Here, separate aliquots of the six samples of total RNAs were prepared and each subjected to ribosomal RNA depletion and subsequent cDNA library construction using a TruSeq Stranded Total RNA with Ribo-Zero Plant kit (Illumina, San Diego, CA). All six samples were then sequenced on the Illumina NextSeq 500 platform as previously described (Al Rwahnih et al., 2018).

### Assembly and Sequence Analysis

The resulting Illumina reads were subjected to adapter trimming and *de novo* assembled into contiguous consensus sequences (contigs) with size lengths of at least 200 base pairs by CLC Bio Genomic Workstation (v8.5.1; Qiagen, Hilden, Germany). These contigs were then annotated by a multistep process for generation of candidate viral sequences as previously described (Al Rwahnih et al., 2018). The first group of contigs was generated with the tBLASTx program (v. 2.4.0) for comparison of sample contig sequences against the viral genome database of the NCBI Refseq (https://www.ncbi.nlm.nih.gov/genome/viruses/) (Tatusova and Madden, 1999). It was determined that any contigs matching viral genomes with a combined E-value equal to or less than 10^-4^ were possible viral candidates.

### Verification of Novel Sequence From HTS Data in ACP Populations

Based on the assembled list of contigs compiled from HTS results, RT-PCR primers were designed to target the 5' and 3' ends of a novel viral sequence detected in HTS. The sequences of the designed primers are shown in **Table 2**. Seasonal representative samples of ACPs from the surveyed sites were then subjected to a new set of RT-PCR analysis for this viral sequence as previously described. This assay was done in order to establish preliminary prevalence of this novel virus in the regional Florida samples as well as to validate our HTS findings.

### Statistical Analysis

For statistical analysis of ACP-associated viruses, the results for the individual county-collected samples were combined into the same six Florida regions that were established for HTS analysis (**Figure 1**). JMP 14 (SAS Corp., USA) statistical software was used to analyze the significant differences between seasonal means of the prevalence of each virus between these six regional Florida samples, comparing adults and nymphs together. Means of the detected presence of each virus in each regional sample were compared to each other using the Student's t test, with significance thresholds set at *P* < 0.05.

## Results

### ACPs Collected From Commercial Citrus Groves

Twenty-one groves, each representing one county in Florida's major citrus producing areas, were identified for surveys. Asian citrus psyllid (ACP) adults and nymphs from each citrus grove were collected monthly (**Figure 1**). During this study, we were able to find and collect a sufficient number of ACP adults and nymphs (n = 25–100ACPs) from each surveyed citrus grove regardless of the collection period (i.e., season). This indicates that ACP overwinter in both adult and nymph forms in Florida citrus groves. The number of ACPs collected, both adults and nymphs, was recorded separately for each grove (representing one county), but for simplicity of data presentation and statistical analyses these numbers were grouped into larger Florida regions (**Table 1** and **Figure 1**). Some monthly-collected county samples lacked collection of either nymph or adult ACP populations, most likely due to inhospitable grove conditions or environmental factors around the time of survey (i.e., recent pesticide application or rain).

### ACP-Associated Virus Screenings With RT-PCR and PCR

**Figure 2:**
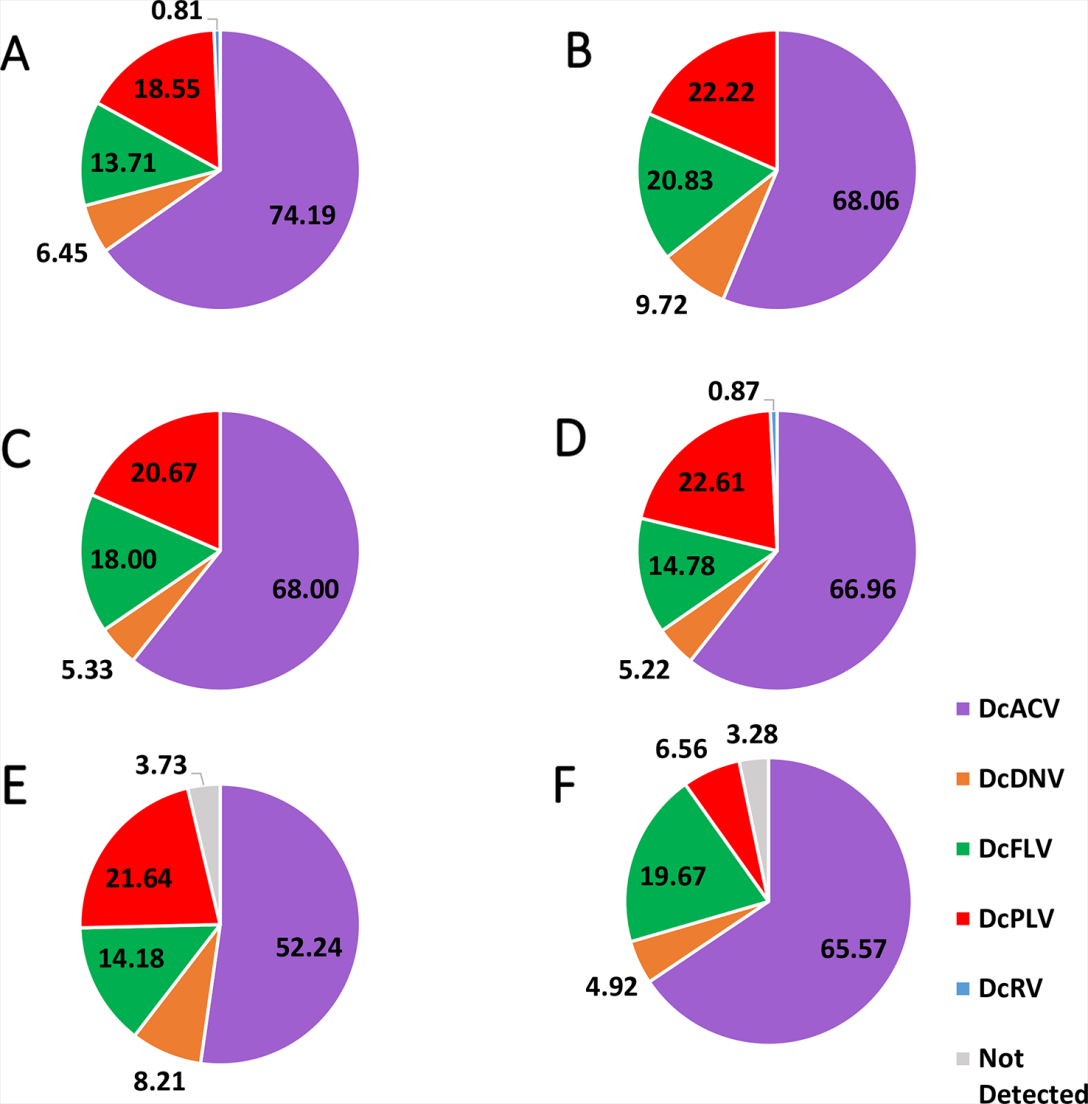
Pie chart showing the percentage of each virus detected in **(A)** Florida Region A, **(B)** Florida Region B, **(C)** Florida Region C, **(D)** Florida Region D, **(E)** Florida Region E, and **(F)** Florida Region F. Note that in the majority of the Florida Regions, there were multiple samples that had multiple viruses; therefore, the majority of the percentages in the pie charts do not equal 100%. Means of the detected percentage of the viruses in each identified region were compared to each other and did not show significant difference (significance threshold set at *P* < 0.05).

Based on our validated virus RT-PCR screenings of ACPs, all of the previously characterized viruses including DcACV, DcFLV, DcDNV, DcRV, and DcPLV were successfully detected in nymph and adult ACP populations collected in Florida citrus groves. Four of the five viruses (DcACV, DcPLV, DcFLV, and DcDNV) were detected in all regions sampled whereas DcRV was detected in only Region A and D (**Table 3** and **Figure 2**). Since viral prevalence showed relative consistency between seasons collected during the study (data not shown), here we present our data showing the more observable differences of viral prevalence between regions (**Figure 2**). The most prevalent virus observed in both nymph and adult ACPs was DcACV (in 67% of the samples) and was detected in every region (**Table 3** and **Figure 2**). The second most prevalent ACP-associated virus detected was the DcPLV, which was detected in 20% of the samples (**Table 3**). Although DcPLV was detected in all regions, its detection was less than DcACV and represented approximately a fifth of the viral detection in each region, except for Region F (**Figure 2**).

**Table 3:** Summary of Florida citrus grove survey results showing the total number of sites (grove/county) surveyed in each region and the number of samples in which ACP-associated viruses were detected in ACPs collected at these sites.

Survey Regions	Total number of samples (adults and nymphs)	Number of sites (grove/county)	Number of samples with *Diaphorina citri*-associated C virus (DcACV)		Number of samples with *Diaphorina citri* flavi-like virus (DcFLV)		Number of Samples with *Diaphorina citri* densovirus (DcDNV)		Number of samples with *Diaphorina citri* reovirus (DcRV)		Number of samples with *Diaphorina citri* picorna-like virus (DcPLV)	
			Adult	Nymph	Adult	Nymph	Adult	Nymph	Adult	Nymph	Adult	Nymph
**Region A**	124	4	47	40	16	7	2	8	0	1	12	16
**Region B**	72	3	32	20	14	2	3	4	0	0	3	10
**Region C**	150	5	56	42	20	11	3	5	0	0	12	17
**Region D**	115	4	39	36	10	12	1	6	1	0	8	17
**Region E**	134	4	44	35	14	8	3	5	0	0	12	12
**Region F**	61	1	28	19	7	5	2	1	0	0	7	2
	**656**	**21**	**Present in all regions (67% of samples)**		**Present in all regions (19% of samples)**		**Present in all regions (7% of samples)**		**Present in 2 regions (<1% of samples)**		**Present in all regions (20% of samples)**	

Each detection of the virus(es) shades the numbered box toward a dark green and represents a simple heat map of prevalence levels for each virus in each region, dark green representing higher levels and dark red representing lower levels.

The DcFLV was found to exist at very close incidence levels to DcPLV (in 19% of the samples) and was detected in both adult and nymph ACPs in all regional Florida samples, though at much lower incidence levels than DcACV; however, it was found to be the third most prevalent virus (**Table 3** and **Figure 2**). The densovirus, DcDNV, was also detected in all regions, but at a much lower incidence level (7% of all samples) than DcFLV or DcPLV (**Table 3** and **Figure 2**). Finally, DcRV was the least prevalent ACP-associated virus detected in less than 1% of the samples and was found in only two of the regions during the entire study (**Table 3** and **Figure 2**).

Incidence levels of DcPLV, DcFLV, DcDNV, and DcRV remained consistently low during the extent of the survey. In contrast, incidence levels of DcACV had a higher level of variation across the regions (**Figure 2**), yet the presence of this RNA virus in ACPs between the different regions was consistently the highest (*P* < 0.05). For example, Region A (**Figure 2A**) and Region E (**Figure 2E**) show DcACV present in these areas, but at different incidence levels throughout the study. DcDNV and DcPLV that were not detected previously in Florida ACP populations (Nouri et al., 2016a) appeared to display a contradicting case during this survey and were actually widespread in Florida. However, DcDNV was found at lower incidence levels than the other viruses (**Figure 2**).

The cDNA samples generated from RNA extracts during the study always tested positive for the ACP internal control, the ACP (*Dc*) wingless gene (data not shown). The ACP wingless gene was used for the confirmation of total RNA and cDNA integrities during this study. The detection of the wingless gene added further confidence in negative samples of the ACP-associated viral survey results.

### Verification of Novel Sequences From HTS Data in ACP Populations

**Figure 3:**
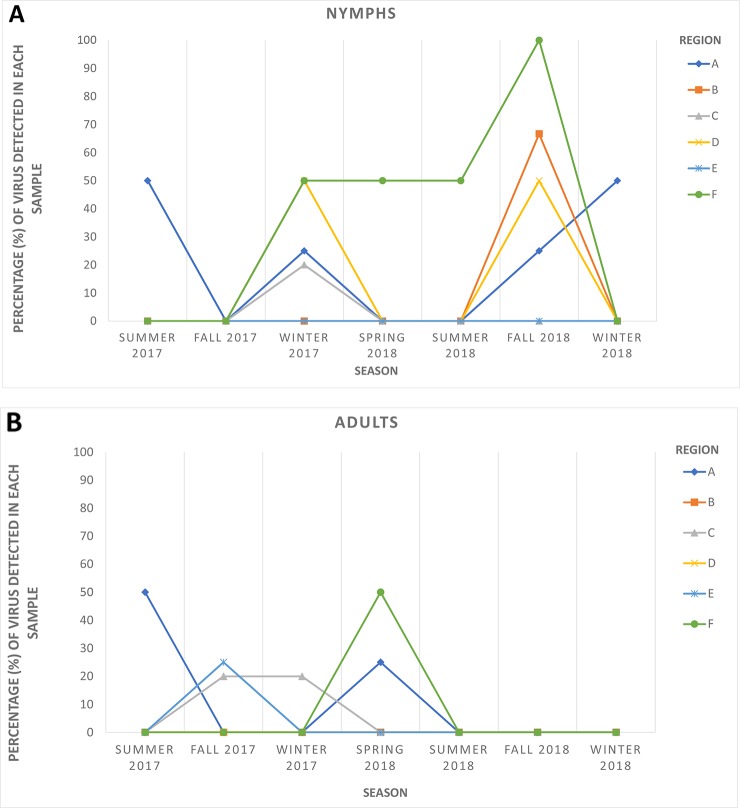
The seasonal prevalence of the novel *Diaphorina citri* cimodo-like virus (DcCLV) in **(A)** nymphs and **(B)** adult ACPs over six seasons (one month per season) in the six larger Florida regions.

HTS yielded between 24 and 35 million raw reads per cDNA library (data not shown). Subsequent analysis identified sequences of several diverse viruses in all ACP samples. As expected, some of these viral sequences shared high similarities with sequences of known ACP-associated viruses, including DcACV and DcFLV. However, there was also a substantial number of sequences sharing similarities with novel viruses that were not known to be present in the ACP (data not shown). For example, a novel virus sequence (~ 4.1 kb in length) with very low similarity (< 50%) to the RNA-dependent RNA polymerase (RdRp) gene of another reovirus from an insect in Africa (Hermanns et al., 2014) was discovered during our HTS analysis. To validate the authenticity and trace back the presence of this virus in our ACP populations collected from the various regions in Florida included in this study, we designed two sets of primers (**Table 2**): one targeting the 5' end of the predicted RdRp and another at the 3' end of the genomic RNA sequence. First, we screened and successfully detected this novel virus throughout the original county samples used for the initial composite sample using RT-PCR with these primers. Simultaneously, seasonal representative samples that were used for the screening of the five known ACP-associated viruses were also used for this novel virus to obtain a general representation of its prevalence (**Figure 3**). This virus, tentatively named *Diaphorina citri* cimodo-like virus (DcCLV), was detected in both adult and nymph ACP populations. It was found in all of the six Florida Regions; however, it excluded most of the seasons in Region B, D, and E (**Figure 3**). Interestingly, we observed a higher incidence of this novel virus in nymph ACP samples than adults (**Figure 3**).

**Figure 4:**
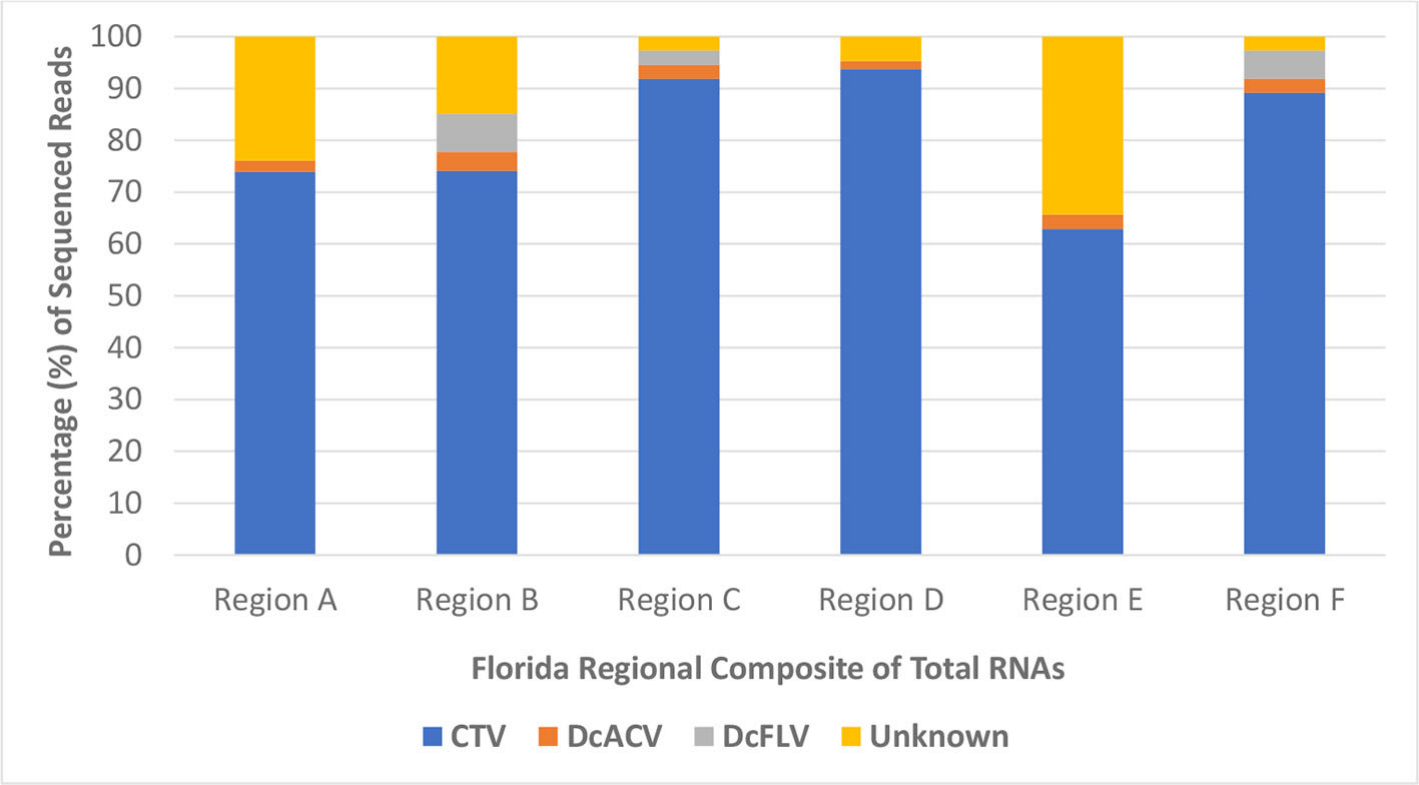
The percentage of high throughput sequencing (HTS) reads per composite sample that matched to a known virus species or were categorized as unknown. CTV, *Citrus tristeza virus*; DcACV, *Diaphorina citri-associated C virus*; DcFLV, *Diaphorina citri flavi-like virus*.

Furthermore, HTS analysis revealed the noticeably higher abundance of *Citrus tristeza virus* (CTV) sRNA sequences in all Florida regional ACP samples compared to any other known virus. More than 60% of all sRNA reads in each regional sample were matched to the CTV genome, with very few detectable reads matching to the DcACV genome (**Figure 4**). Only half of the regional samples had any DcFLV sRNA reads detected, and as expected, all of the samples had levels of unknown sRNA reads, which supports our hypothesis of novel viruses present in the ACP. Interestingly, an additional novel picornavirus, with 77% sequence similarity to *Graminella nigifrons virus* 1 (Chen et al., 2015), was also detected in Region A (data not shown). Yet, for all of the regions, CTV composed at least 60% of all HTS reads and suggests possible uptake of CTV by the ACP from infected trees during phloem-feeding.

## Discussion

To achieve the goal of this study's first objective, we collected thousands of nymph and adult ACPs from 21 of Florida's major citrus-producing counties in order to effectively survey and monitor the diversity of viruses in the ACP microbiome (**Table 1** and **Figure 1**). Five previously identified ACP-associated viruses, DcACV, DcFLV, DcDNV, DcRV, and DcPLV were chosen from prior literature investigating the ACP virome, applied to this survey study, and successfully detected and monitored in these populations over approximately eighteen consecutive months (six seasons). Although these viruses were previously found in ACPs from other countries, only three of them (DcACV, DcFLV, and DcRV) were reported in ACP populations from Florida (Marutani-Hert et al., 2009; Matsumura et al., 2016; Nouri et al., 2016a; Nouri et al., 2016b). However, our survey results indicate that all five viruses were widespread and present at varying incidences in ACP populations throughout Florida citrus groves (**Table 3** and **Figure 2**). To the best of our knowledge, this is the first study that has conducted a comprehensive and grove-based survey throughout Florida for establishing the prevalence levels of these viruses and the identification of two new viruses in ACP populations since their introduction into the state in 1998.

Our survey results indicate that the DcACV was detected at a higher level than any of the other four ACP-associated viruses in nymph and adult ACP populations collected throughout Florida citrus groves. And, DcACV RNAs were also detected in partially purified virions from nymph and adult ACPs (data not shown). This, along with the greater variance of DcACV incidence levels for all regions, shows the need for quantification of these viruses in future studies to compare how abundant they are in the ACP. DcFLV and DcPLV were also detected throughout most of the sampled citrus-producing counties, although not every month as was seen with DcACV, which suggests these viruses may exist at lower titers in the ACPs of these regions. Surprisingly, we also detected DcDNV throughout these vector populations, which has not been observed in Florida before this study (Nouri et al., 2016a). Remarkably, although Nouri et al. (2016a) did not detect the DNA virus DcDNV or the RNA virus DcPLV in their ACP samples from Florida, it was detected in ACPs throughout all Florida regions surveyed (**Table 3** and **Figure 2**). This finding supports the novel presence of this ACP-associated virus in Florida and adds increased diversity to the list of potential viral vectors to pursue for biological control. Finally, the extremely low incidence levels of DcRV detected in our study seemed to contrast with the >50% incidence level reported by Marutani-Hert et al. (2009). Even this information on the lack of viral detection in these collected ACP samples can strengthen the case of the diversity and fluctuation of the ACP virome within the same region of citrus groves sampled. Nonetheless, all five of the viruses in this study were detected in both adult and nymph ACP populations, which suggests an infectious and/or propagative nature of these viruses passing from adult female ACPs to nymphs.

RNAi defense mechanisms represent an integral part of the insect immune response to viral infections and allows for the substantial detection of viral sRNAs among total insect RNA extracted (El-Shesheny et al., 2013; Roossinck et al., 2015; Pecman et al., 2017). The insect vector of the HLB pathosystem, the ACP, also has these resistance capabilities, and was analyzed extensively during this study to further understand its virome as it relates to Florida citrus groves (Van Rij, 2011; Hall et al., 2013; Taning et al., 2016). Since the detection and prevalence of these five previously identified ACP-associated viruses allowed for only a piece of the ACP virome puzzle, we simultaneously compiled early ACP RNA samples of all county sites into six regional samples and subjected them to HTS technologies. Each of the six composite RNA samples were based on the combined regional counties (Region A-F) shown in **Figure 1**. When we applied HTS technologies toward analysis of these insects, the sequenced and analyzed results of the RNA samples revealed a frozen snapshot of the viral composition and identity of the Florida ACP virome, as shown in **Figure 4**. Although this analysis only represents a small subset of ACP populations in Florida citrus groves between August 2017 and May 2018, when combined with the results of our simultaneously conducted PCR and RT-PCR survey screenings, it provides substantial evidence to the varying levels of viral prevalence detected. Not only does this further support the high prevalence of DcACV in Florida ACP populations, but also suggests that it might be well adapted to this insect host.

However, it should be noted that our survey results show the detection of each ACP-associated virus when originating from a five-insect subset specimen (composite sample) of each monthly-collected county sample. This consistent number of insects for each population was intended to standardize possible incidence levels detected of each virus. If subsets of ACPs used for RNA extraction and cDNA synthesis were increased, incidences of these viruses may change and show different prevalence levels.

The second objective of the current study was to examine populations of ACP from Florida citrus groves through HTS of small RNAs in an attempt to discover additional unknown viruses associated with this insect. Such metagenomic approaches with similar goals have been successfully implemented to discover diverse and novel viruses from field-collected mosquitoes, ACPs, *Drosophila* flies, and various other types of insects (Marutani-Hert et al., 2009; Ma et al., 2011; Cook et al., 2013; Coffey et al., 2014; Webster et al., 2015; Matsumura et al., 2016; Nouri et al., 2016a; Nouri et al., 2016b). A desirable translational outcome of this objective would be to identify ACP-associated viruses that have the potential to be used as biological agents to control *D. citri* and halt the spread of HLB, particularly in the endemic environment of Florida. In this study, we were able to identify and assemble nearly complete genome sequences of several putative novel viruses associated with ACP, including a novel reo-like virus and a picornavirus. To survey the newly identified reo-like virus, which is tentatively named *Diaphorina citri cimodo-like virus* (DcCLV), in six Florida regional ACP populations, specific primers were designed based on the fragments obtained from bioinformatics analysis and used to screen additional *D. citri* populations, which were not analyzed by HTS (**Table 2** and **Figure 3**). The DcCLV was detected in both adult and nymph ACP populations and was found in all six Florida regions, although at varying prevalence levels (**Figure 3**). However, we observed a higher incidence of this novel virus in nymph ACP samples than adults, which may suggest a plausible lethality to the ACP as infected nymphs may not survive to adulthood. Nevertheless, this hypothesis must be substantiated with more evidence gathered through further studies.

One of the unexpected outcomes of the HTS results was the discovery that CTV, a non-ACP-associated virus, is extremely abundant in the Florida ACP virome (**Figure 4**). To further validate this finding, we went back and screened representative cDNA samples of our ACP populations collected during early months of this study using RT-PCR with CTV-specific primers. We were able to detect this virus in both adult and nymph ACP samples collected throughout Florida citrus groves, as well as in subsequently purified additional virion samples from ACPs by immunogold labelling with CTV-specific antibodies using transmission electron microscopy (data not shown). Together these results cautiously suggest that although CTV is not causing noticeable decline or showing visible symptoms in citrus trees, the virus persists throughout Florida. The fact that non-pathogenic CTV-RNAi vectors are currently being deployed into the citrus host and our discovery of CTV persistence in Florida citrus groves calls for further scrutiny into possible cross-reactivity of viral strains (Hajeri et al., 2014). Unanswered questions regarding what CTV may be doing in the ACP warrants more studies. Future research into these molecular interactions may provide deeper insight into the uncertainty of CTV's modified deployment in the citrus host (Harper and Cowell, 2016). Furthermore, the detection of a novel picornavirus with high (77%) sequence similarities with *Graminella nigifrons virus 1*, a picornavirus known to be associated with leafhoppers (Chen et al., 2015), in our samples also deserves future inquiry into reasons for its presence in the ACP.

Comprehensive and grove-based viral surveys during this study (i.e., RT-PCR and PCR) and multiple sRNA read detections using HTS established that DcACV was the most prevalent and widespread ACP-associated virus in Florida citrus groves. Following the completion of this study, our specific approach for targeting the mortality of the ACP as a biological control will undoubtedly influence the choice of viral vector we will venture forth with. On the one hand, our research findings provide robust evidence for DcACV as the likely choice to pursue as a possible viral candidate in RNA interference technology. Its clear ubiquity deserves future research into possible utilization as an RNAi delivery machinery against the HLB vector. On the other hand, the low prevalence and detection of DcRV may be considered as unfavorable to the ACP and could serve as an even better viral candidate for RNAi delivery. Regardless of the virus' abundance, any modified viral vector will require high durability in the insect to avoid losing efficacy, as it will be constantly subjected to evolutionary changes and potential suppression by the ACP. Detection of all the viruses in both nymph and adult ACP samples suggest the potential of vertical transfer of each virus from parent to progeny and each virus' capability to remain in the ACP populations. DcACV's consistent detection and prevalence in ACPs and its prevalence in detection through HTS also suggests the highest stability observed amongst the viruses during this study in the ACP virome. DcACV may not be treated as a foreign entity if manipulated and re-inoculated into wild Florida ACP populations, which strengthens its potential as an RNAi vector tool for future studies.

In conclusion, this comprehensive survey and the successful detection of two novel and five previously characterized ACP-associated viruses, coupled with further HTS analysis of these Florida ACP populations, have provided innovative and clearer insight regarding the spatial and temporal prevalence of these viruses in natural grove conditions. These results have greatly increased our knowledge of a potential source of viral candidates to use against this economically devastating insect vector in Florida. To capitalize on this study, our next steps will include establishing the presence and significance of *C*Las in these same ACP RNA samples used for the ACP-associated viral surveys. We also hope to determine the precise tissue localization of these ACP-associated viruses in the ACP, which will help strengthen future research by determining how efficient and effective this viral candidate's RNAi capabilities could be toward controlling the HLB disease cycle by killing the ACP.

## Data Availability Statement

All datasets generated for this study are included in the article**/**supplementary material.

## Author Contributions

KB performed the experiments, collected ACPs, analysed the data and drafted the manuscript. SG helped with RT-PCRs and PCRs, ACP collections, statistical analysis, and editing the manuscript. MR completed all the HTS. OB and AL received the funds, designed and directed the study. OB identified grove sites, collected ACP, purified virions, analysed the data and wrote the manuscript. All authors reviewed and edited the manuscript.

## Funding

Funding for this project was provided by the Citrus Initiative Grant of University of Florida IFAS and U.S. Department of Agriculture (USDA) under grant number 2015-70016-23011.

## Conflict of Interest

The authors declare that the research was conducted in the absence of any commercial or financial relationships that could be construed as a potential conflict of interest.
